# Odor Fingerprint Analysis Using Feature Mining Method Based on Olfactory Sensory Evaluation

**DOI:** 10.3390/s18103387

**Published:** 2018-10-10

**Authors:** Hong Men, Yanan Jiao, Yan Shi, Furong Gong, Yizhou Chen, Hairui Fang, Jingjing Liu

**Affiliations:** 1 Advanced Sensor Technology Institute, College of Automation Engineering, Northeast Electric Power University, Jilin 132012, China; 2201600437@neepu.edu.cn (Y.J.); 2201500430@neepu.edu.cn (Y.S.); 2201700354@neepu.edu.cn (F.G.); fanghairui@neepu.edu.cn (H.F.); 2 Department of Neurobiology and Behavior, University of California, Irvine, CA 92697, USA; yizhouc1@uci.edu

**Keywords:** odor fingerprint analysis, feature mining method, olfactory sensory evaluation, time domain, frequency domain, intelligent nose, Chinese liquor

## Abstract

In this paper, we aim to use odor fingerprint analysis to identify and detect various odors. We obtained the olfactory sensory evaluation of eight different brands of Chinese liquor by a lab-developed intelligent nose. From the respective combination of the time domain and frequency domain, we extract features to reflect the samples comprehensively. However, the extracted feature combined time domain and frequency domain will bring redundant information that affects performance. Therefore, we proposed data by Principal Component Analysis (PCA) and Variable Importance Projection (VIP) to delete redundant information to construct a more precise odor fingerprint. Then, Random Forest (RF) and Probabilistic Neural Network (PNN) were built based on the above. Results showed that the VIP-based models achieved better classification performance than PCA-based models. In addition, the peak performance (92.5%) of the VIP-RF model had a higher classification rate than the VIP-PNN model (90%). In conclusion, odor fingerprint analysis using a feature mining method based on the olfactory sensory evaluation can be applied to monitor product quality in the actual process of industrialization.

## 1. Introduction

Due to its particularity and generality, fingerprint can provide the basis to distinguish between samples due to its uniqueness and reliability [1]. Odor fingerprint analysis is preferred to the use of intelligent instruments which are sensitive to the stimulation of odor to produce the relevant data of volatile feature components. Adoption of odor fingerprint analysis is widely used in the field of foods. For example, the maturity of fruits could be expressed by the odor intensity [2], the degree of freshness [3], and diseases [4,5] could be determined by odor fingerprint analysis. Thus, the use of odor as a biometrics recognition method is feasible [6].

Chinese liquors belong to the distilled liquor which is loved by people for its strong aromatic odor. As a traditional fermented beverage, the saccharifying ferment of Chinese liquor is daqu, xiaoqu, bran koji and yeast wine, which is produced with cereal grains as the main raw materials and is processed by distilling, saccharifying and fermenting [7]. The microconstituents of liquors are organic compounds which directly influence the flavor of liquor quality. These organic contents are 1% to 2% including acids, esters, alcohols, aldehydes, and so on. Depending on the different brewing techniques and raw materials, different liquors have significant differences in aromatic characters and odor fingerprint. Therefore, the case study of Chinese liquor is representative and typical.

Currently, there are multiple studies on liquors by traditional sensory evaluation and physical or chemical methods [8,9]. With the advantages of simple operation and immediate results, the sensory evaluation method is generally acknowledged and widely adopted. However, this method mainly depends on the reviewers’ subjective sense of smell and taste [10]. In addition, there is a great deal of time and cost to train qualified professionals. On the other hand, the physical or chemical method analyzes samples using infrared spectrometer, chromatographic analyzer, mass spectrometer and other instruments [11,12]. These methods are reliable to analyze constituents of samples. However, there are some shortcomings, such as time-consuming, costs. These methods cannot comprehensively evaluate sample and cannot meet the development of process of industrialization.

Along with the rapid development of artificial intelligence, intelligent nose, also known as artificial nose, intelligent olfaction system, as a new bionic detection instrument, could make up the deficiency of the traditional olfactory sensory evaluation [13]. Intelligent nose consists of three parts: sensor array, signal processing unit, and pattern recognition. These three parts can respectively simulate the acquisition of information by human olfactory receptor sensory neurons, the encoding of the olfactory nerve, and the processing of information by the human olfactory system [14]. Therefore, odor fingerprint information gathered by intelligent nose could evaluate samples comprehensively. Based on the human olfaction, each olfactory neuron can detect different odorant molecules. On the other hand, each odorant molecule is able to respond to multiple olfactory neurons. The same goes for the principle of intelligent nose: Different sensors respond differently to different odorants. The intelligent nose can provide overall information of volatile compounds and it is widely used in analyzing the quality of wine [15], tobacco [16], tea [17], rice [18] and fruit [19,20,21]. Furthermore, it also involved in medical diagnosis [22], environment monitoring [23] and other fields. However, there are only a handful of published studies focusing on the detection of odor fingerprint information of Chinese liquors using intelligent nose, such as analyzing different flavor types [24], authenticity [25], place origin [26] and age [27].

As is known to all, drift is an inevitable question in measurement. Sensor drift refers to the output of sensor changes from time to time when the input remains unchanged. Currently, it is believed that sensor drift is caused by two causes, on the one hand is the chemical process which occurs between the sensor material and the environment, on the other hand is the system noise [28]. In practice, the outputs gradually fail to match the right gases for sensor drift reason. For this problem, researchers have done a great deal of work to ensure the response of sensors. Ma et al. [28] proposed the ODAELM-S and ODAELM-T for online sensor drift compensation in E-Nose systems. This method aims to achieve timely processing without losing the recognition accuracies for sensor drift. Zhang et al. [29] proposed the DAELM-S and DAELM-T to compensate sensor drift. Another effective method is unsupervised feature adaptation (UFA)-based transfer, learning ideas for enhancing the drift tolerance of E-noses [30]. The above methods focus on online compensation to resolve the sensor drift effectively.

In this paper, taking Chinese liquors as an example, we followed the offline sensor drift compensation approach for the intelligent nose system while the majority of past studies have focused on the simulation of human olfaction to detect and identify odor fingerprint information.

The appropriate multivariate statistical and pattern recognition methods can effectively increase the differentiation of odor fingerprints based on the intelligent nose and can check the accuracy of models. Previous research primarily focused on the feature extraction of time domain features such as peak, mean, maximum variance, root mean square and standard deviation [31,32]. However, the basic characteristics of signals, both in the time domain and frequency domain, can provide comprehensive angles for signal analysis. The time domain is the only real domain which is parallel to the real world and it is the relationship between mathematical functions and physical signals to time. While the frequency domain is a mathematical category which follows particular rules which can reveal the inner characteristics signals [33,34]; the feature extraction method, combining time domain and frequency domain features, can be used to mine information that reflects different odor fingerprint features about samples [35,36]. However, this method causes information redundancy, that is, as the number of dimensions increases, the training time and forecasting time of the model will take longer. Therefore, it is of greatest importance to find a more reasonable and effective feature mining method to extract efficient features.

**Figure 1:**
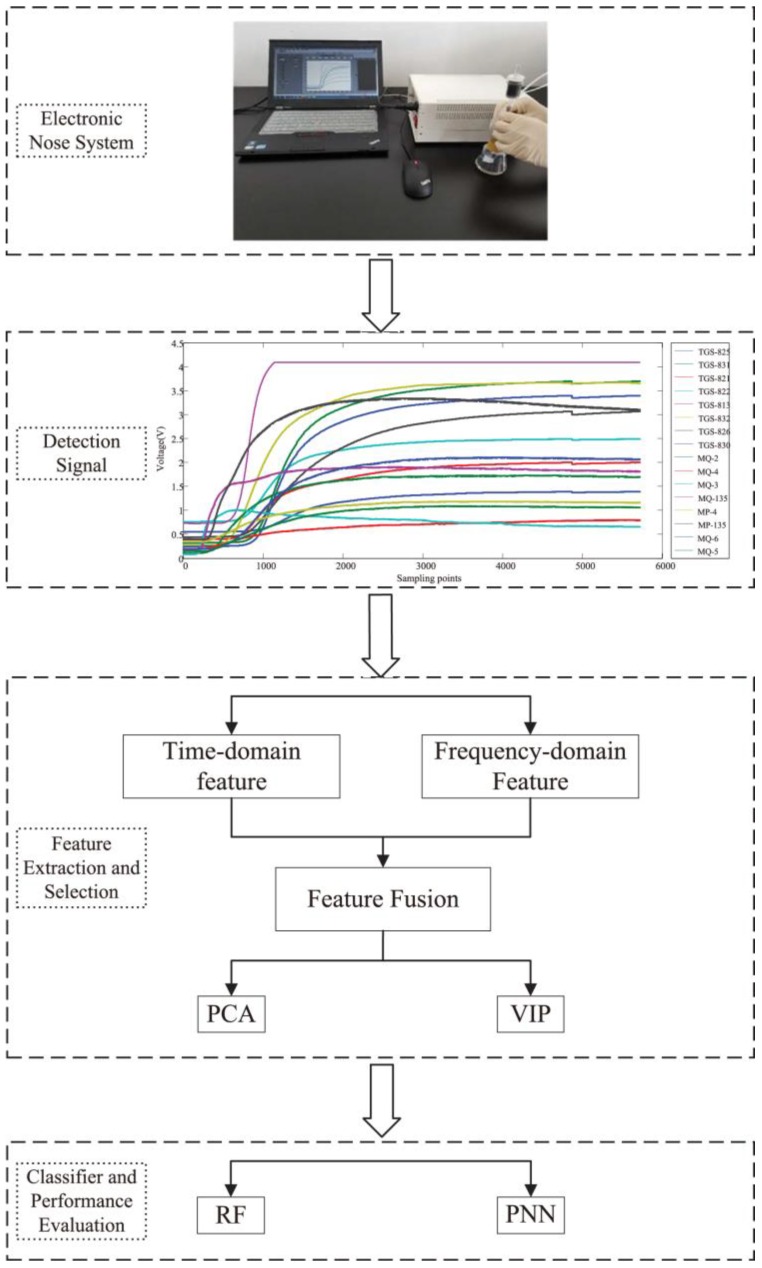
Flow chart of odor fingerprint analysis.

Taking eight different brands of Chinese liquors as an example, this paper aims to use the odor fingerprint analysis, simulate human olfaction through experiments with the lab-developed intelligent nose and adopt the feature mining method to detect and identify various odors. According to the raw experimental data from 16 sensors of the lab-developed intelligent nose, we extracted the time domain and frequency domain characteristics to construct the odor fingerprint. In addition, odor fingerprints were analyzed by PCA and VIP scores for selecting characteristic features. Next, we selected Random Forest (RF) and Probabilistic Neural Network (PNN) to dynamically characterize the interactions among the feature variables, and then obtained the best variable characteristics and the highest classification accuracy. This is a significant study for the detection and identification of Chinese liquors through odor fingerprint analysis based on the olfactory sensory evaluation. Figure 1 shows the flow chart of odor fingerprint analysis for this article.

## 2. Materials and Methods

### 2.1. Liquor Samples

In this paper, eight different brands of Chinese liquors purchased at a local liquor store were selected as samples. These samples differed in brand, alcohol content, flavor, raw materials, and origin. Details were listed in Table 1.

### 2.2. Intelligent Nose

**Figure 2:**
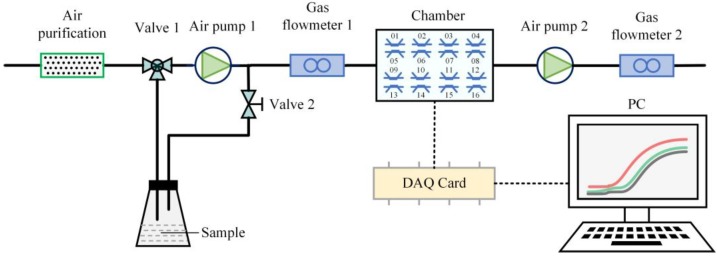
The block diagram of the intelligent nose analysis system.

As shown in Figure 2, the lab-developed intelligent nose system contains three units—the air flow velocity and direction control unit (consists of air purification, valve, gas flowmeter, and air pump), the sensors unit (includes sensor arrays and chamber), and the data acquisition and analysis unit (contains data acquisition card (DAQ) and PC with the self-made test software). The two major functions (gas injection and system cleaning) were carried out by adjusting valves. The air purification consists of activated carbon, molecular sieve and allochroic silicagel gel, and more remarkably, allochroic silicagel gel, which belongs to the high-grade drying agent, can visually signal the relative humidity of the environment according to the color variation (from blue to red). It is usually used for instruments, equipments and other closed conditions. The role of air pump 1 and 2 are to clean the system and to collect gas, respectively. In addition, the combination of these two air pumps are used to raise the gas volume rate in the gas cleaning process. The dimension of the chamber is 10.5 cm long, 8.2 cm wide and 5 cm high with a volume of about 431 cm^3^. The chamber is made of cardboard which is covered by Polytetrafluoroethylene (PTFE). PTFE has weak adsorption and strong leakproofness so that there is no other interfering research to affect the test results in the air chamber. Sensor arrays contain a temperature sensor, humidity sensor and 16 independent sensors. LM35CZ type temperature sensor by National Semiconductor, Santa Clara, CA, USA and HIH-4000-003 type humidity sensor by Yi Jiajie Electronic Technology CO., LTD, ShenZhen, China, in the air chamber are used to monitor the internal temperature and humidity. Sixteen independent sensors are sensitive to different substances. These sensors can detect odor fingerprint data and consist of two systems: TGS-8 system by FIGARO, Japan and MQ/MP system by ZhengZhou Winsen Electronics Techbology CO., LTD, ZhengZhou, China. Details of these sensors used in the experiment are listed in Table 2. The NI USB-6211 type data acquisition card by National Instruments, Austin, TX, USA, was selected to collect data. There are eight analog input channels and two analog output channels and the sample rate reaches 48 Ks/s.

The static head-space sampling method was adopted in this experiment. The lab environment is best to control the temperature at 23 ± 2 °C and the relative humidity at 60 ± 5%. The experimental procedure was performed as following:

(1) Open the air pump 2 and valve 1, put a clean and empty Erlenmeyer flask in the defined location. Then observe the zero value of each sensor and compare with the standard value.

(2) Twenty milliliters of the sample was put in a 100 mL Erlenmeyer flask, sealed and left to sit for 5 min.

(3) Close air pump 2 and adjust air pump 1 so that the gas flowmeters 1 and 2 (by Qihai Electromechanical Manufacturing CO., LTD, Chengdu, China) display 2 L/min to clean windpipes for 10 s. Then open air pump 2 to clean the entire device. This process lasted 5 min to eliminate the influence by other gases.

(4) Place the test samples in the defined location and adjust air pumps 1 and 2 so that the gas flowmeters 1 and 2 display 0.5 L/min to let the gas enters the chamber. Ten seconds later, close air pump 2 and keep the gas coming into the chamber sequentially. At the same time, observe the signals and record test data.

(5) Without loss of generality, repeat the experiment 10 times for each sample by repeating Steps (2)–(4). Note that the relative humidity will not change in the course of the experiment. At last, a total of 80 sets of data is obtained.

In this paper, we extracted time domain and frequency domain features to construct an odor fingerprint map. The time-domain feature is the average value (AV) of intelligent nose response signals of sensors. The frequency domain feature is the mean of variance (MV) of the eight wavelet packet coefficients obtained by three layers of wavelet packet decomposition with db6 wavelet [37].

The time domain features of the *i*th sensor of TGS-8 system were defined as:

(1) $${AV}_{Ti}=\frac{x_{Ti1}+x_{Ti2}+\ldots \ldots +x_{Ti5940}}{5940}(i=1,2,\ldots \ldots ,8)$$

where *x_Ti_*_1_, *x_Ti_*_2_, …, *x_Ti_*_5940_ are response value of the *i*th sensor of TGS-8 system intelligent nose.

The time domain features of the *i*th sensor of the MQ/MP system were defined as:

(2) $${AV}_{Mi}=\frac{x_{Mi1}+x_{Mi2}+\ldots \ldots +x_{Mi5940}}{5940}(i=1,2,\ldots \ldots ,8)$$

where *x_Mi_*_1_, *x_Mi_*_2_, …, and *x_Mi_*_5940_ are response values of the *i*th sensor of the MQ/MP system electronic nose.

The time domain features of the *i*th sensor of the TGS-8 system were defined as:

(3) $${MV}_{Ti}=\frac{S_{Ti1}+S_{Ti2}+\ldots \ldots +S_{Ti8}}{8}(i=1,2,\ldots \ldots ,8)$$

where *S_Ti_*_1_, *S_Ti_*_2_, …, *S_Ti_*_8_ are the variance yields extracted from the coefficients of the wavelet packet of the *i*th sensor of the TGS intelligent nose; the response value measured from the intelligent nose was decomposed into wavelet packet components based on the db6 wavelet, and then extracting the coefficients of the wavelet packet.

The frequency domain features of the *i*th sensor of the MQ/MP system were defined as:

(4) $${MV}_{Mi}=\frac{S_{Mi1}+S_{Mi2}+\ldots \ldots +S_{Mi8}}{8}(i=1,2,\ldots \ldots ,8)$$

where *S_Mi_*_1_, *S_Mi_*_2_, …, *S_Mi_*_8_ in the formula are the variance yields extracted from coefficients of wavelet packet of the *i*th sensor of the MQ/MP intelligent nose; the response value measured from the intelligent nose was decomposed into wavelet packet components based on the db6 wavelet, and then extracting the coefficients of the wavelet packet.

### 2.3. Feature Selection

#### 2.3.1. Data Processing of Odor Fingerprint Analysis

As is known to all, sensor sensitivity has a great influence on the intelligent nose system performance. The sensitivity of the sensor should be considered to achieve the best performance.

**Figure 3:**
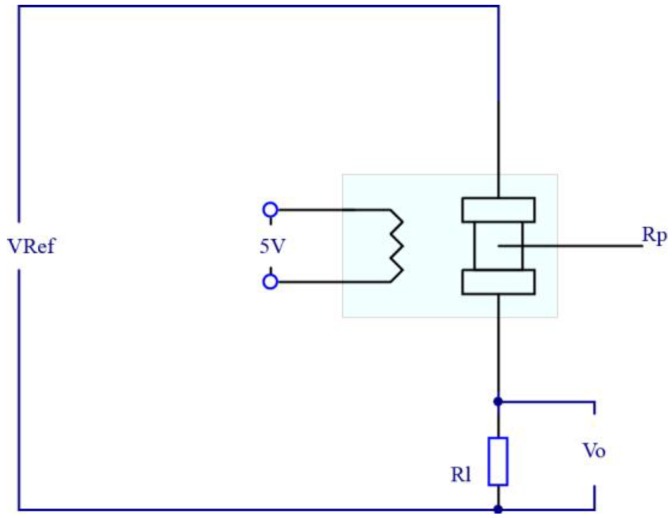
Drive circuit of the sensor.

As shown in Figure 3, in the drive circuit of the sensor, *R_p_* is the resistance value of the sensor. *R_l_* is the resistance value of the load resistance and the output voltage of sensor is the voltage across the load resistance. The relationship between the output voltage and reference voltage is as follows:

(5) $$V_{o}=V_{Ref}\cdot R_{l}/(R_{p}+R_{l})$$

$$\begin{array}{l} ∵V_{o}=V_{Ref}\cdot R_{l}/(R_{p}+R_{l})\\ ∴\Delta V_{o}=-R_{l}\cdot V_{Ref}\cdot \Delta R_{p}/{\left(R_{p}+R_{l}\right)}^{2}\\ ∴\left|\Delta V_{o}/\Delta R_{p}\right|=V_{Ref}\cdot R_{l}/{\left(\sqrt{R_{l}}+R_{p}/\sqrt{R_{l}}\right)}^{2} \end{array}$$

$$∵{\left(\sqrt{R_{l}}+R_{p}/\sqrt{R_{l}}\right)}^{2}\geq 2R_{p}$$

All these show that when *R_l_* is equal to *R_p_*, the sensor has the greatest response sensitivity to improve the performance of the intelligent nose system.

**Figure 4:**
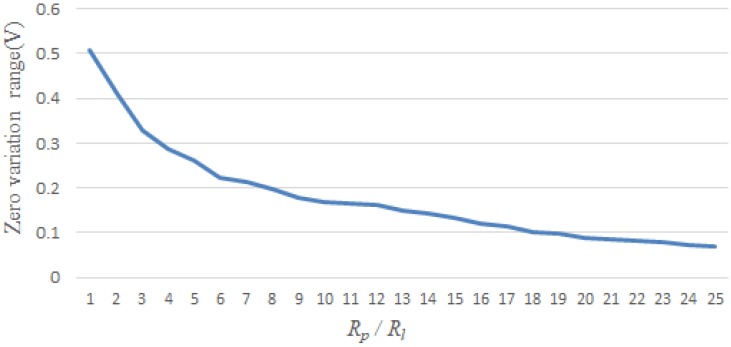
TGS-821 sensor’s zero value changes with *R_l_* variation.

As shown in Figure 4, taking the TGS-821 sensor for example, the best output response was studied by changing different *R_l_* values. Other sensors have the same characteristics.

**Figure 5:**
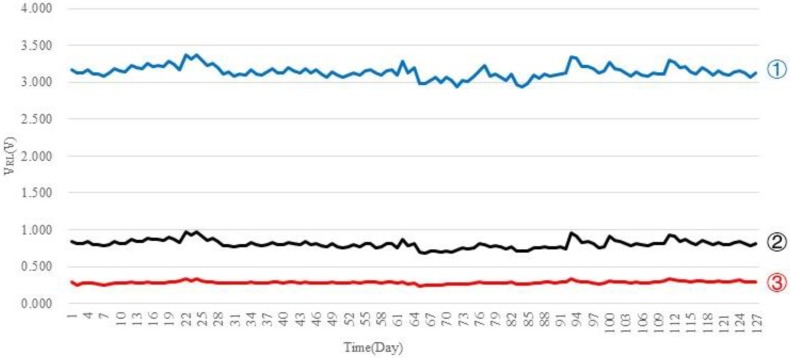
Data graph of sensors. ①.*R_l_* = *R_p_*, ② *R_l_* = (1/10)*R_p_*, ③ *R_l_* = (1/16)*R_p_*.

As shown in Figure 5, in order to find the appropriate resistance value of the load resistor in experiment, we perform an experiment with the purpose of supervising the zero value of sensors which continued for 127 days. By experiment, when *R_l_* is about one-fifteenth of the value of *R_p_*, the output response of sensors is obvious.

Signal processing, as an important step of improving the performance of the intelligent nose, refers to preprocess signals of sensor array responses. The standardized processing is the most popular method that translates raw data into a dimensionless index. Therefore, this step can avoid pattern recognition failure because of the large magnitude of some sensors. We choose the relative difference method to suppress sensor drift. *x_s_*(0) is the zero response value of the sensor.

(6) $$y_{s}(t)=\frac{x_{s}(t)-x_{s}(0)}{x_{s}(0)}$$

Then, in order to expedite the convergence rate of the model, the odor fingerprint information obtained by different sensors should be converted to the same dimension and the same order of magnitudes. We normalized the fusion feature sets and the normalized interval is (0, +1). After the series of the above-mentioned processing (relative difference method and normalization), additive drift and response drift of the sensor will be suppressed.

**Figure 6:**
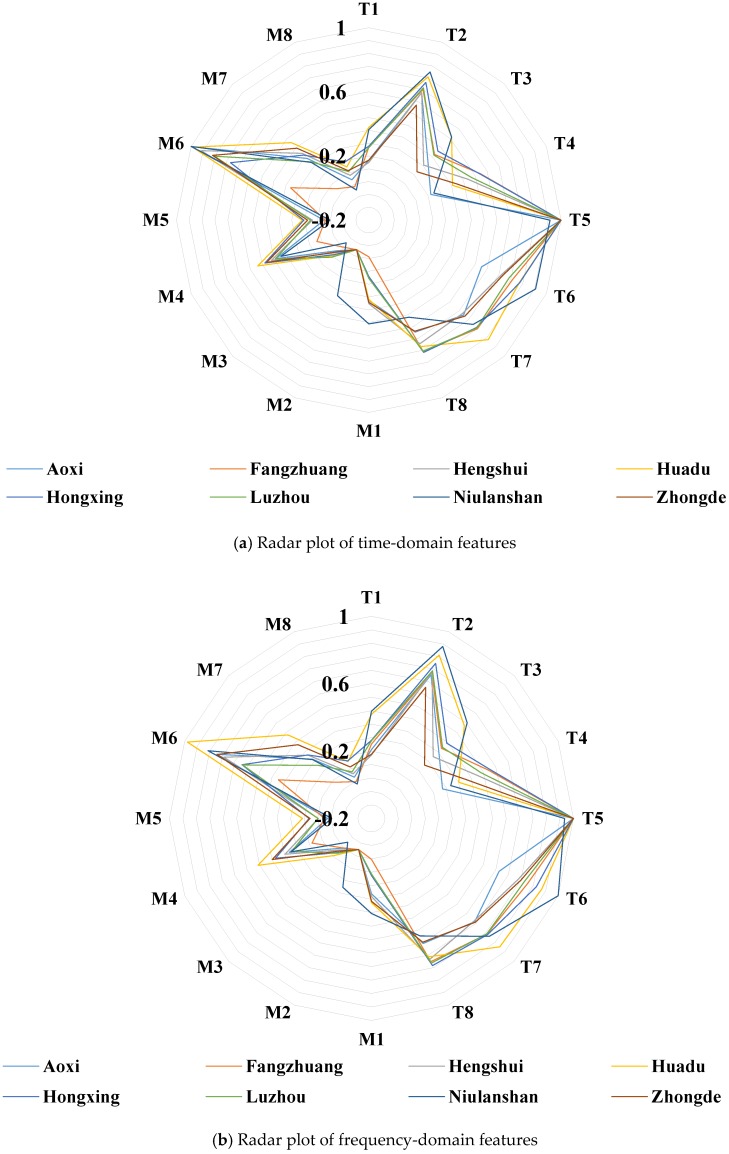
Radar plot for different kinds of Chinese liquors.

Figure 6a,b shows radar plots for time domain and frequency domain features, respectively. Since each sensor detects cross-information of olfactory, it is difficult to determine which features are the characteristic values that affect the olfactory information of liquors. It can be seen that the sensor T3 and T4 are obviously different in AV value. Does it proves these two values are the main factors affecting the olfactory information of Chinese liquors? Meanwhile, the sensor M1 and M2 are slightly difference in MV value. Does it proves these two characteristics have little effect on the olfactory information of Chinese liquors? Therefore, it is indispensable to find a suitable feature mining method to delete the redundant information and select characteristic features that can affect the olfactory information of Chinese liquors. In addition, the best combination of variables and fusion methods to reduce the complexity of the model prediction and achieve the best classification performance have to be chosen.

#### 2.3.2. Feature Extraction and Filtering

Principal Component Analysis (PCA) is a meaningful multivariate statistical method. It can convert multiple variables to a few comprehensive variables through linear transforming. These comprehensive variables that are principal components can reflect most of the information of the original variables at the greatest extent. These principal components are not only linearly independent of each other but also mutually orthogonal. In this paper, PCA was used to process the original features that fused the time domain and frequency domain. From this, principal components can express characteristic features of Chinese liquors’ olfactory information.

In the Partial Least Square (PLS), the Variable Importance of Projection (VIP) scores were used to create a new data space in a lower dimensional system [38]. The VIP scores can express the interpretative ability of the independent variables to dependent variables. With higher scores meaning a greater rate of contribution to covariance and stronger distinguishing ability, each variable of the original feature was evaluated and obtained corresponding scores. These variables were sorted based on the VIP scores and selected to form the new characteristic space. The feature fusion strategy is as follows: (1) The original features that fused time domain and frequency domain were sorted based on VIP scores. (2) K = [k_1_, k_2_, …, k_m_] variable subsets were generated based on the best VIP scores. Which ki means the subset has top *i*th variables and m is the number of all variables. In this paper, we analyzed the original features and generated 32 subsets based on the VIP scores to express the interaction between different variables.

#### 2.3.3. Multivariate Analysis

In this paper, altogether 80 sets of data were divided into two parts based on the Kennard-stone algorithm, 1/2 as the training set and the rest as the testing set. The former was used to construct the classification model and the latter was used to test the classification performance of models established by the former.

The KS algorithm is commonly used as an effective method to select a training set. In the KS algorithm, all samples were considered as candidates for training sets that were selected in order. The KS algorithm can be summarized as follows: (1) Calculating the distance between every two samples and selecting the two samples with the largest distance. (2) Calculating the distance between the remaining sample and the selected two samples, respectively. (3) Repeating this step until the number of selected samples is equal to the predetermined number [39].

Random Forest (RF) is an ensemble of classification and regression tree (CART). It was first proposed by Kam in 1995 [40] and Breiman made an intensive study [41]. The essence of RF is a nonlinear classifier that contains multiple decision trees. There is no correlation between these trees. When the testing data entered into the random forest, the data was classified by each decision tree. The final results are the most classified results in all trees.

With its fast training rate and simple realization, it is widely used in biological information [42], ecology [43], medicine [44], economic finance [45], computer vision [46], speech [47], data mining [48], remote sensing geography [49] and other fields. The execution procedure of RF is: Assuming that the number of attributes of the sample is M. Resampling based on the Bootstrap method. Then T training sets *S*_1_, *S*_2_, …, *S_T_* were generated. (2) The corresponding decision trees C_1_, C_2_, …, C_T_ were generated by each training set. Before the property was selected on each internal node, m properties that were randomly selected from M properties should be seen as the split attribute set of the current node. (3) Each tree has complete growth without pruning. (4) For the testing set sample X, every decision tree was tested to obtain the corresponding categories C_1(X)_, C_2(X)_, …, C_T(X)_. (5) By taking the vote, the most output category in the T decision trees was taken as the category of the testing set.

Probabilistic Neural Networks (PNN) is the supervised classifier which was first put forward by D. F. Speeht in 1990 [50]. It is a parallel algorithm based on the Bayes classification rule and the Parzen window’s probability density function. With its simple learning process, fast training speed, better compatibility and strong nonlinear ability, PNN was applied to image recognition [51], chemical detection [52] and stereo vision matching [53] fields. PNN generally consists of four layers: The input layer, the model layer, the summation layer, and the output layer. The steps of PNN networks are as follows: (1) Collecting sample data and dividing into a training set and a testing set. (2) Creating PNN networks and training the network according to training sets. (3) Testing network performance.

## 3. Results

### 3.1. Dimension Reduction by PCA

**Figure 7:**
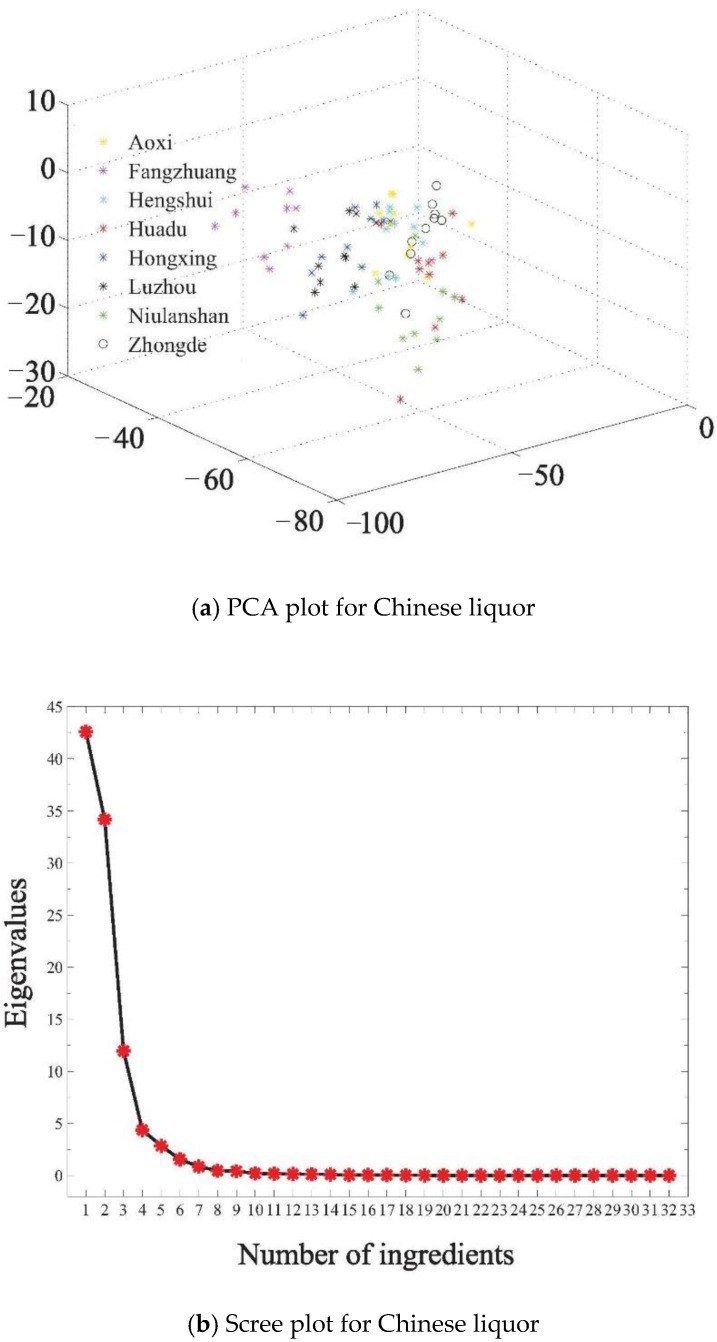
PCA scatter plot for Chinese liquor.

The odor fingerprint information obtained in the experiment was analyzed by the PCA algorithm. The first three principal components account for 42.59%, 34.16%, and 11.95% respectively. Figure 7a shows the PCA processing results of different brands of Chinese liquors.

Observing the scree plot from Figure 7b, when the number of principal components reaches 10, the polyline area is stable. The cumulative contribution of principal components reaches 99.368%, which can represent all characteristic data. Therefore, we extracted the first 10 principal components as a new feature data set to substitute the original variables. Results showed that it provides a reliable method to construct a little more concise odor fingerprint map.

### 3.2. Variable Selection by VIP Scores

**Figure 8:**
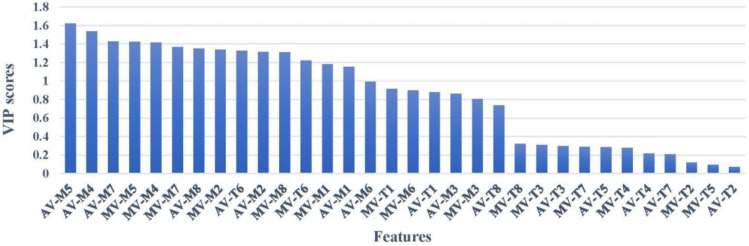
Relative variable importance based on calculated VIP.

Figure 8 shows the VIP scores for each feature variable of the original fusion dataset measured by PLS discrimination analysis. As shown, the VIP score of AV_M5_, AV_M4_, AV_M7_, MV_M5_, MV_M4_, MV_M7_, AV_M8_, MV_M2_, AV_T6_, AV_M2_, MV_M8_, MV_T6_, MV_M1_ and AV_M1_ are greater than 1, indicating that these variables have significant meaning in the odor fingerprint of Chinese liquors. While the VIP scores of the rest are less than 1, which means that these variables have less effect on the classification of Chinese liquors, VIP scores cannot give a verdict for the classification performance of models. Therefore, we found a series of fusion matrix as an input of the model based on VIP scores. Each subset includes the top several variables, in other words, subset #1 includes AV_M5_, subset #2 contains AV_M5_ and AV_M4_, the last subset #32 contains all variables. We can select the prime variable combination by dynamically observing the classification performance of RF networks and PNN network. Results showed that it provides a reliable method to construct a much more concise odor fingerprint map by selecting the best combination of variables.

Table 3 shows the accuracy rate achieved by RF and PNN models. As the number of variables increases, the classification accuracy rates show an upward tendency. Specifically, the classification accuracy of RF and PNN in subset #11 have reached the same accuracy as the original fusion dataset. This indicates that the original fusion dataset contains a large amount of redundant information. With the number of variables increasing, RF models appeared to have the highest accuracy rate of 92.5% under the subset #15 and PNN appeared to have the highest accuracy rate of 87.5% under subset #16. We continued to raise variables, and the accuracy rate of each model did not exceed the above-mentioned maximum value. These results are consistent with the VIP scores shown in Figure 8. That is, the performance of the model increased with variables added whose VIP scores were greater than one, while the performance of the model decreased with the rest of the variables added whose VIP scores were less than one. From above, we chose subset #15 as the best combination.

### 3.3. Classification Using Random Forest

In RF networks, the value of mtry and the number of decision trees are the main parameters of generalization performance. The default mtry value is the square root of the total number of variables, so the value of mtry in the experiment was four. We selected the number of decision trees from 2 to 100 at two trees intervals. The training accuracy rate and predicting accuracy rate were regarded as the evaluation criterion. From this, we can focus on the influence of decision trees on the classification performance in RF networks.

**Figure 9:**
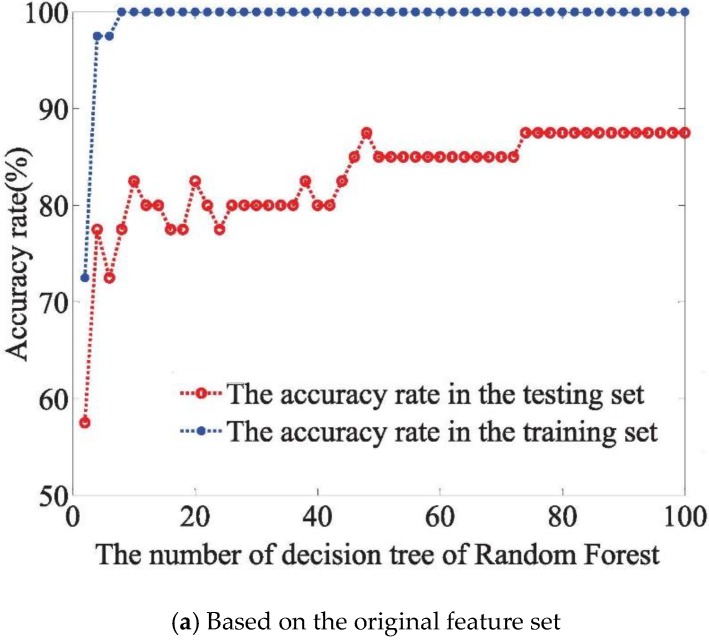

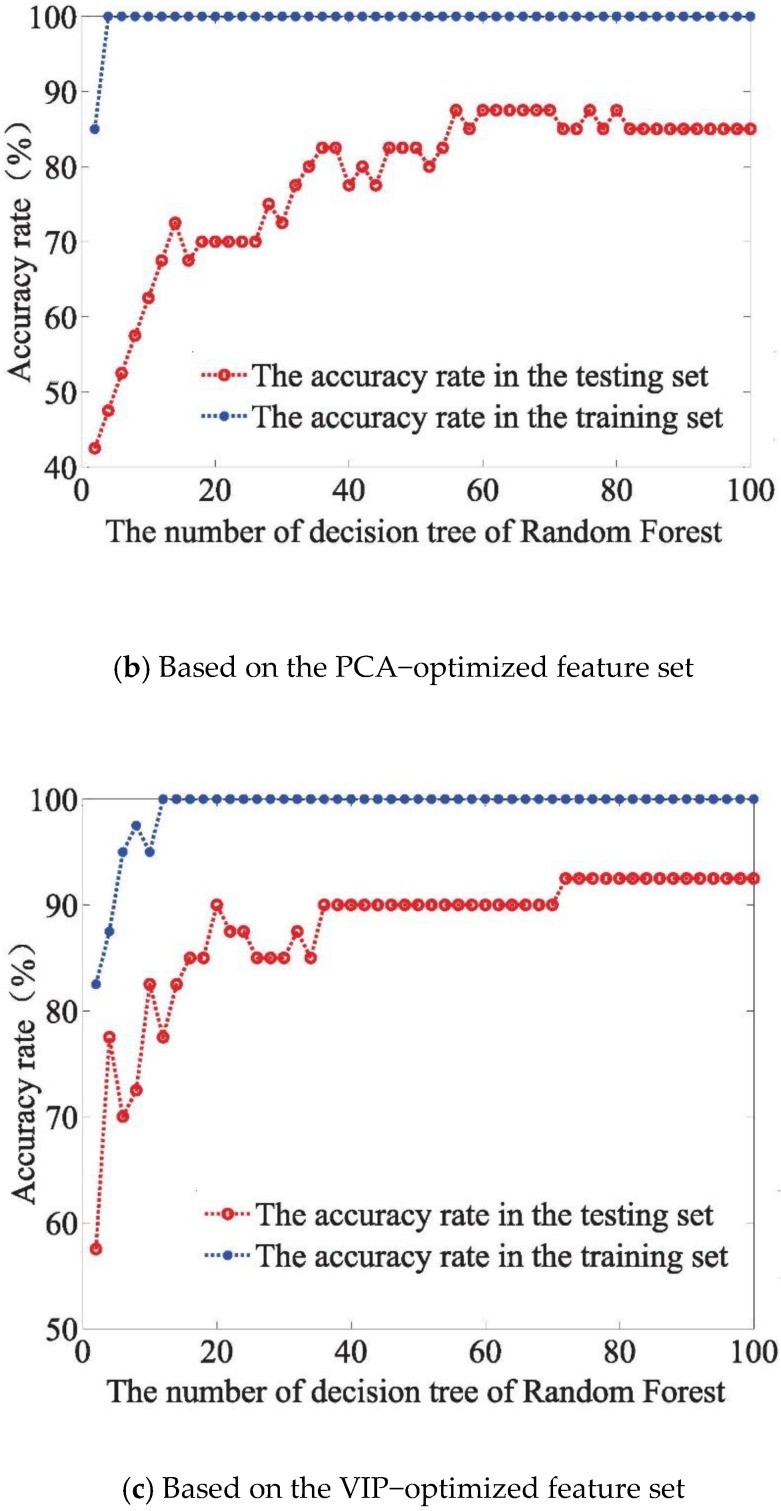
Classification performance of RF network based on decision trees.

The three feature sets (original, PCA-optimized, and VIP-optimized, from which the 15th variable subset was extracted based on the VIP scores) combined with the RF model achieved the classification for olfactory information of Chinese liquors. To reduce the impact of randomness, 100 prediction models were established, and their accuracy rates were averaged as the classification accuracy rate of the current model. As shown form Figure 9a–c, the training accuracy rate reaches 100% when the number of decision trees is greater than 8, 4, and 12, respectively. Besides, in the RF model based on the VIP-optimized feature set, when the number of decision trees exceeds 72, the testing accuracy reaches up to 92.5%. Further, along with the continual increase of the decision trees, the system remains stable. Results showed that the olfactory information of original features contains redundant information. Besides, the feature mining method based on VIP-optimized can extract effective features.

### 3.4. Classification Using PNN

**Figure 10:**
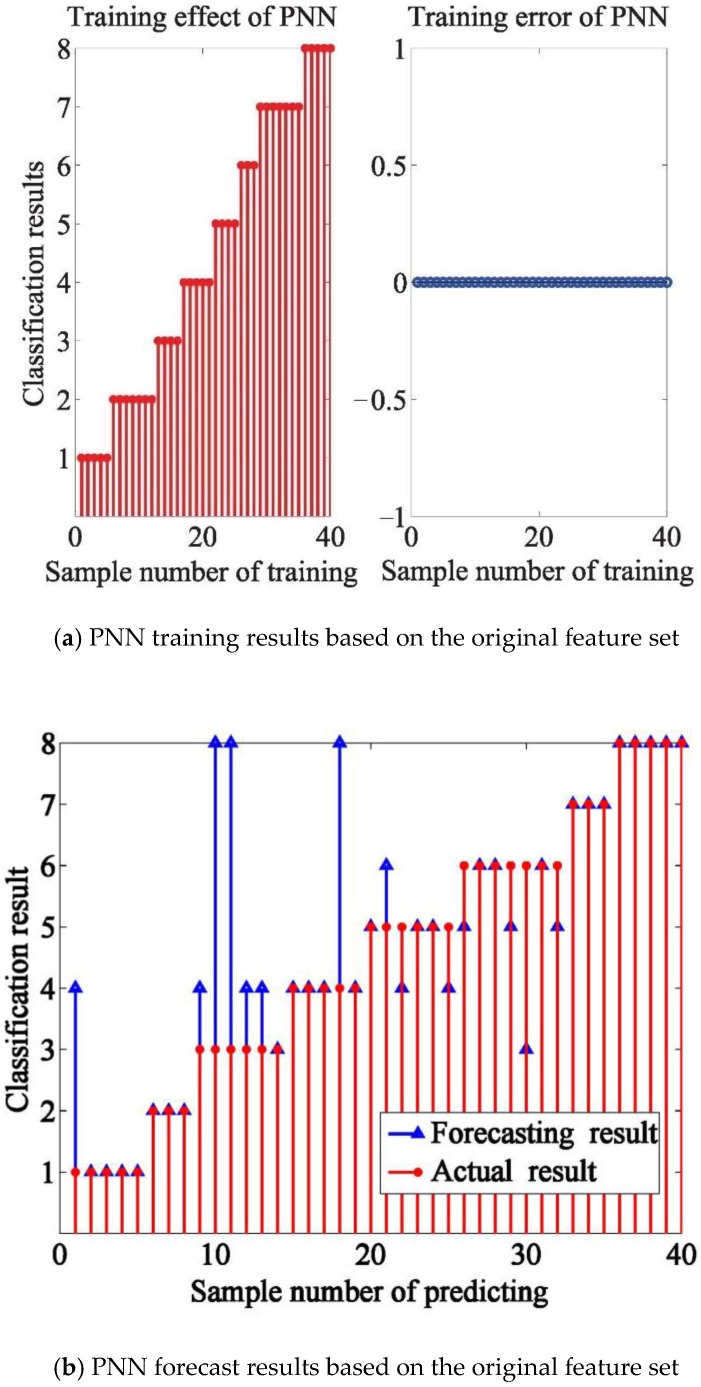

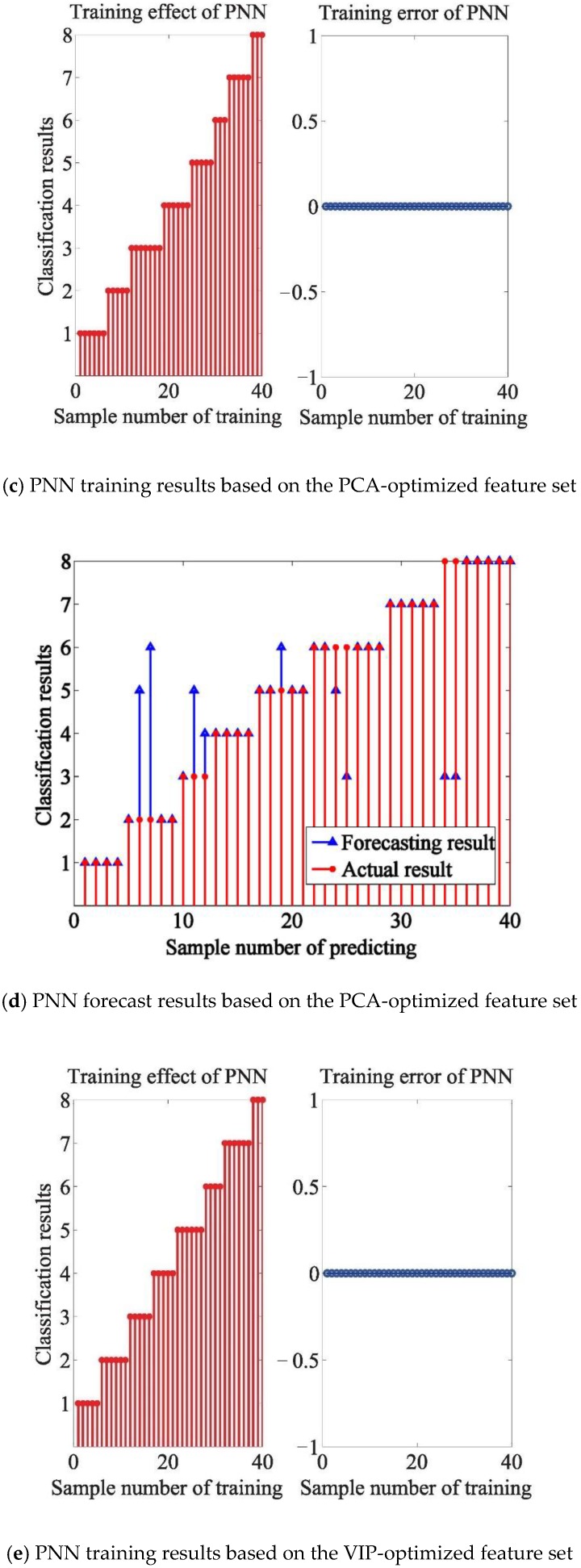

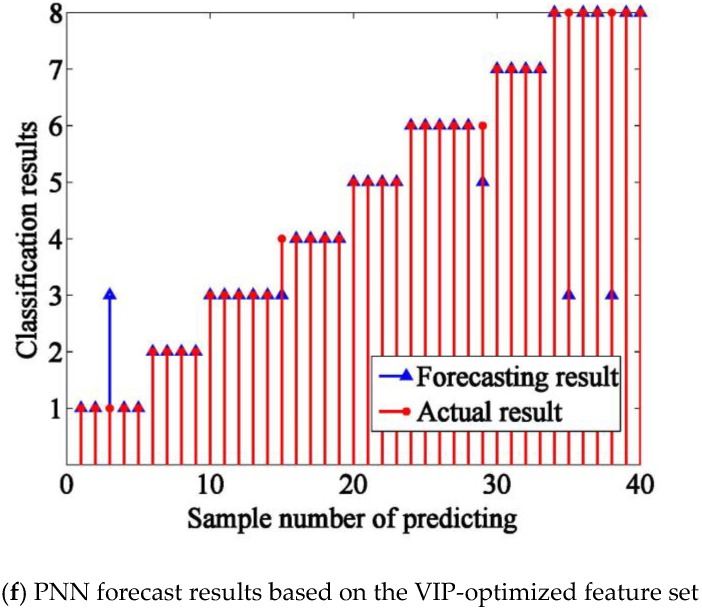
Classification performance of PNN network based on PNN.

The three feature sets (original, PCA-optimized, and VIP-optimized from which the 16th variable subset was extracted based on the VIP scores) combined with the PNN model work well in classifying the olfactory information of Chinese liquors. As shown in Figure 10a,c and e, 40 training samples were classified correctly, as shown in predicting effect of PNN, the test accuracy rate was 65%, 77.5% and 87.5% with 40 test samples (The vertical axis is category label. And from 1 to 8 are category labels of eight brands of Chinese liquors, respectively.).

The PNN models based on PCA-optimized and VIP-optimized are superior to the model based on the original features, which means that there is a lot of redundant information in the original features. Compared with the PNN model based on PCA-optimized, the model of VIP-optimized performed well, which means that the feature mining method based on VIP-optimized can improve the accuracy rate and extract effective features.

## 4. Discussion

Table 4 shows the classification accuracies under different data processing and pattern recognition methods. As shown in Table 4:

(1) By comparison, the classification accuracy of the RF network was better than the PNN network based on the different feature methods. Thus, it can be seen that the RF network has stronger processing power in this experiment.

(2) Compared with the original features, classification performance did not significantly improve based on the PCA-optimized both in the RF network and PNN network. The data processing method based on PCA cannot obtain the best combination of variables to identify various odors more accurately.

(3) Compared with the original feature and PCA-optimized, selected features based on the best VIP scores obtained the obvious promotion of the classification performance. The classification accuracy of the RF network in subset #15 and the PNN network in subset #16 was 92.5% and 87.5%, respectively. Finally, the RF network showed the best classification performance of 92.5% in subset #15. Combined with VIP scores, AV_M5_, AV_M4_, AV_M7,_ MV_M5_, MV_M4_, MV_M7_, AV_M8_, MV_M2_, AV_T6_, AV_M2_, MV_M8_, MV_T6_, MV_M1_, AV_M1_, and AV_M6_ were considered as the characteristic features.

## 5. Conclusions

In conclusion, taking eight different brands of Chinese liquors as an example, our work adopted the odor fingerprint analysis based on olfactory sensory evaluation and the feature mining method which combined the time domain and frequency domain to simulate human olfaction and to identify various odors. Variable selection using VIP scores is especially suitable for extracting features from a mass of data. In addition, the VIP-based models achieved better prediction accuracies than the PCA’s. The results demonstrated that VIP coupled with the RF or PNN network is effective in extracting and analyzing features of odor fingerprint. Compared with the PNN model, the RF model achieved the slightly higher accuracy. Meanwhile, compared with the traditional statistical methods and simple extraction, this feature mining method used the least characteristic variables and the best fusion method and can capture hidden patterns and variables inside the odor fingerprint. The odor fingerprint analysis using the feature mining method based on olfactory sensory evaluation can be applied to the food and drinks industry for product discrimination, classification, quality and control. Besides, the lab-developed intelligent nose can be used in the actual process of industrialization to monitor product quality.

## Figures and Tables

**Table 1:** Liquor sample characteristics.

No.	Brand	Alcohol Content (%vol)	Flavor Type	Main Raw Material	Place of Origin
1	Aoxi Erguotou	56	Feng-flavor	pure water, Chinese sorghum	Tongzhou district, Peking City
2	Fangzhuang Beijing Erguotou	56	Feng-flavor	pure water, red sorghum	Daxing district, Peking City
3	Hengshui old white dry	50	Laobaigan-flavor	Chinese sorghum, wheat, pure water	Hengshui City, Hebei Province
4	Huadu Beijing Erguotou	56	Feng-flavor	pure water, Chinese sorghum	Changping district, Peking City
5	Hongxing Erguotou	56	Feng-flavor	Chinese sorghum, pure water, corn, barley, pea	Jixian county, Tianjin
6	Luzhou Laojiao	45	Luzhou-flavor	pure water, Chinese sorghum, wheat	Luzhou city, Sichuan Province
7	Niulanshan Erguotou	56	Feng-flavor	pure water, Chinese sorghum, barley, wheat, pea	Shunyi district, Peking City
8	Zhongde Erguotou	43	Feng-flavor	pure water, Chinese sorghum, wheat	Fangshan district, Peking City

**Table 2:** Characteristics of sensors.

No.	Sensor Name	Sensitive Gas	Detection Range (mg/L)
1	TGS-825	Hydrogen sulfide	5–100
2	TGS-831	R-21 and R-22	100–3000
3	TGS-821	hydrogen	30–1000
4	TGS-822	Ethanol	50–5000
5	TGS-813	Methane, Propane and Butane	500–10,000
6	TGS-832	R-134a	100–3000
7	TGS-826	Ammonia	30–300
8	TGS-830	R-113, hydrogen and Ethanol	100–3000
9	MQ-2	Ethanol, Propane and hydrogen	300–10,000
10	MQ-4	Alkanes	300–10,000
11	MQ-3	Ethanol	40–4000
12	MQ-135	Hydrogen, R-113 and Ethanol	10–1000
13	MP-4	Methane	300–10,000
14	MP-135	hydrogen	30–1000
15	MQ-6	Isobutane, Propane and LPG	300–10,000
16	MQ-5	Methylpropane	300–10,000

**Table 3:** Comparison of the results based on different classification models.

Subsets	Features	RF (%)	PNN (%)
#1	AV_M5_	35	27.5
#2	AV_M5_ + AV_M4_	60	35
#3	AV_M5_ + AV_M4_ + AV_M7_	72.5	35
#4	AV_M5_ + AV_M4_+AV_M7_ + MV_M5_	67.5	47.5
#5	AV_M5_ + AV_M4_ + AV_M7_ + MV_M5_ + MV_M4_	72.5	72.5
#6	AV_M5_ + AV_M4_ + AV_M7_ + MV_M5_ + MV_M4_ + MV_M7_	77.5	60
#7	AV_M5_ + AV_M4_ + AV_M7_ + MV_M5_ + MV_M4_ + MV_M7_ + AV_M8_	70	62.5
#8	AV_M5_ + AV_M4_ + AV_M7_ + MV_M5_ + MV_M4_ + MV_M7_ + AV_M8_ + MV_M2_	85	82.5
#9	AV_M5_ + AV_M4_ + AV_M7_ + MV_M5_ + MV_M4_ + MV_M7_ + AV_M8_ + MV_M2_ + AV_T6_	85	80
#10	AV_M5_ + AV_M4_ + AV_M7_ + MV_M5_ + MV_M4_ + MV_M7_ + AV_M8_ + MV_M2_ + AV_T6_ + AV_M2_	85	80
#11	AV_M5_ + AV_M4_ + AV_M7_ + MV_M5_ + MV_M4_ + MV_M7_ + AV_M8_ + MV_M2_ + AV_T6_ + AV_M2_ + MV_M8_	87.5	75
#12	AV_M5_ + AV_M4_ + AV_M7_ + MV_M5_ + MV_M4_ + MV_M7_ + AV_M8_ + MV_M2_ + AV_T6_ + AV_M2_ + MV_M8_ + MV_T6_	80	67.5
#13	AV_M5_ + AV_M4_ + AV_M7_ + MV_M5_ + MV_M4_ + MV_M7_ + AV_M8_ + MV_M2_ + AV_T6_ + AV_M2_ + MV_M8_ + MV_T6_ + MV_M1_	85	60
#14	AV_M5_ + AV_M4_ + AV_M7_ + MV_M5_ + MV_M4_ + MV_M7_ + AV_M8_ + MV_M2_ + AV_T6_ + AV_M2_ + MV_M8_ + MV_T6_ + MV_M1_ + AV_M1_	85	60
#15	AV_M5_ + AV_M4_ + AV_M7_ + MV_M5_ + MV_M4_ + MV_M7_ + AV_M8_ + MV_M2_ + AV_T6_ + AV_M2_ + MV_M8_ + MV_T6_ + MV_M1_ + AV_M1_ + AV_M6_	92.5	80
#16	AV_M5_ + AV_M4_ + AV_M7_ + MV_M5_ + MV_M4_ + MV_M7_ + AV_M8_ + MV_M2_ + AV_T6_ + AV_M2_ + MV_M8_ + MV_T6_ + MV_M1_ + AV_M1_ + AV_M6_ + MV_T1_	82.5	87.5
#17	AV_M5_ + AV_M4_ + AV_M7_ + MV_M5_ + MV_M4_ + MV_M7_ + AV_M8_ + MV_M2_ + AV_T6_ + AV_M2_ + MV_M8_ + MV_T6_ + MV_M1_ + AV_M1_ + AV_M6_ + MV_T1_ + MV_M6_	85	87.5
#18	AV_M5_ + AV_M4_ + AV_M7_ + MV_M5_ + MV_M4_ + MV_M7_ + AV_M8_ + MV_M2_ + AV_T6_ + AV_M2_ + MV_M8_ + MV_T6_ + MV_M1_ + AV_M1_ + AV_M6_ + MV_T1_ + MV_M6_ + AV_T1_	87.5	87.5
#19	AV_M5_ + AV_M4_ + AV_M7_ + MV_M5_ + MV_M4_ + MV_M7_ + AV_M8_ + MV_M2_ + AV_T6_ + AV_M2_ + MV_M8_ + MV_T6_ + MV_M1_ + AV_M1_ + AV_M6_ + MV_T1_ + MV_M6_ + AV_T1_ + AV_M3_	87.5	87.5
#20	AV_M5_ + AV_M4_ + AV_M7_ + MV_M5_ + MV_M4_ + MV_M7_ + AV_M8_ + MV_M2_ + AV_T6_ + AV_M2_ + MV_M8_ + MV_T6_ + MV_M1_ + AV_M1_ + AV_M6_ + MV_T1_ + MV_M6_ + AV_T1_ + AV_M3_ + MV_M3_	85	82.5
#21	AV_M5_ + AV_M4_ + AV_M7_ + MV_M5_ + MV_M4_ + MV_M7_ + AV_M8_ + MV_M2_ + AV_T6_ + AV_M2_ + MV_M8_ + MV_T6_ + MV_M1_ + AV_M1_ + AV_M6_ + MV_T1_ + MV_M6_ + AV_T1_ + AV_M3_ + MV_M3_ + AV_T8_	85	82.5
#22	AV_M5_ + AV_M4_ + AV_M7_ + MV_M5_ + MV_M4_ + MV_M7_ + AV_M8_ + MV_M2_ + AV_T6_ + AV_M2_ + MV_M8_ + MV_T6_ + MV_M1_ + AV_M1_ + AV_M6_ + MV_T1_ + MV_M6_ + AV_T1_ + AV_M3_ + MV_M3_ + AV_T8_ + MV_T8_	87.5	65
#23	AV_M5_ + AV_M4_ + AV_M7_ + MV_M5_ + MV_M4_ + MV_M7_ + AV_M8_ + MV_M2_ + AV_T6_ + AV_M2_ + MV_M8_ + MV_T6_ + MV_M1_ + AV_M1_ + AV_M6_ + MV_T1_ + MV_M6_ + AV_T1_ + AV_M3_ + MV_M3_ + AV_T8_ + MV_T8_ + MV_T3_	85	67.5
#24	AV_M5_ + AV_M4_ + AV_M7_ + MV_M5_ + MV_M4_ + MV_M7_ + AV_M8_ + MV_M2_ + AV_T6_ + AV_M2_ + MV_M8_ + MV_T6_ + MV_M1_ + AV_M1_ + AV_M6_ + MV_T1_ + MV_M6_ + AV_T1_ + AV_M3_ + MV_M3_ + AV_T8_ + MV_T8_ + MV_T3_ + AV_T3_	82.5	72.5
#25	AV_M5_ + AV_M4_ + AV_M7_ + MV_M5_ + MV_M4_ + MV_M7_ + AV_M8_ + MV_M2_ + AV_T6_ + AV_M2_ + MV_M8_ + MV_T6_ + MV_M1_ + AV_M1_ + AV_M6_ + MV_T1_ + MV_M6_ + AV_T1_ + AV_M3_ + MV_M3_ + AV_T8_ + MV_T8_ + MV_T3_ + AV_T3_ + MV_T7_	82.5	77.5
#26	AV_M5_ + AV_M4_ + AV_M7_ + MV_M5_ + MV_M4_ + MV_M7_ + AV_M8_ + MV_M2_ + AV_T6_ + AV_M2_ + MV_M8_ + MV_T6_ + MV_M1_ + AV_M1_ + AV_M6_ + MV_T1_ + MV_M6_ + AV_T1_ + AV_M3_ + MV_M3_ + AV_T8_ + MV_T8_ + MV_T3_ + AV_T3_ + MV_T7_ + AV_T5_	82.5	77.5
#27	AV_M5_ + AV_M4_ + AV_M7_ + MV_M5_ + MV_M4_ + MV_M7_ + AV_M8_ + MV_M2_ + AV_T6_ + AV_M2_ + MV_M8_ + MV_T6_ + MV_M1_ + AV_M1_ + AV_M6_ + MV_T1_ + MV_M6_ + AV_T1_ + AV_M3_ + MV_M3_ + AV_T8_ + MV_T8_ + MV_T3_ + AV_T3_ + MV_T7_ + AV_T5_ + MV_T4_	77.5	67.5
#28	AV_M5_ + AV_M4_ + AV_M7_ + MV_M5_ + MV_M4_ + MV_M7_ + AV_M8_ + MV_M2_ + AV_T6_ + AV_M2_ + MV_M8_ + MV_T6_ + MV_M1_ + AV_M1_ + AV_M6_ + MV_T1_ + MV_M6_ + AV_T1_ + AV_M3_ + MV_M3_ + AV_T8_ + MV_T8_ + MV_T3_ + AV_T3_ + MV_T7_ + AV_T5_ + MV_T4_ + AV_T4_	77.5	67.5
#29	AV_M5_ + AV_M4_ + AV_M7_ + MV_M5_ + MV_M4_ + MV_M7_ + AV_M8_ + MV_M2_ + AV_T6_ + AV_M2_ + MV_M8_ + MV_T6_ + MV_M1_ + AV_M1_ + AV_M6_ + MV_T1_ + MV_M6_ + AV_T1_ + AV_M3_ + MV_M3_ + AV_T8_ + MV_T8_ + MV_T3_ + AV_T3_ + MV_T7_ + AV_T5_ + MV_T4_ + AV_T4_ + AV_T7_	80	67.5
#30	AV_M5_ + AV_M4_ + AV_M7_ + MV_M5_ + MV_M4_ + MV_M7_ + AV_M8_ + MV_M2_ + AV_T6_ + AV_M2_ + MV_M8_ + MV_T6_ + MV_M1_ + AV_M1_ + AV_M6_ + MV_T1_ + MV_M6_ + AV_T1_ + AV_M3_ + MV_M3_ + AV_T8_ + MV_T8_ + MV_T3_ + AV_T3_ + MV_T7_ + AV_T5_ + MV_T4_ + AV_T4_ + AV_T7_ + MV_T2_	87.5	70
#31	AV_M5_ + AV_M4_ + AV_M7_ + MV_M5_ + MV_M4_ + MV_M7_ + AV_M8_ + MV_M2_ + AV_T6_ + AV_M2_ + MV_M8_ + MV_T6_ + MV_M1_ + AV_M1_ + AV_M6_ + MV_T1_ + MV_M6_ + AV_T1_ + AV_M3_ + MV_M3_ + AV_T8_ + MV_T8_ + MV_T3_ + AV_T3_ + MV_T7_ + AV_T5_ + MV_T4_ + AV_T4_ + AV_T7_ + MV_T2_ + MV_T5_	87.5	65
#32	AV_M5_ + AV_M4_ + AV_M7_ + MV_M5_ + MV_M4_ + MV_M7_ + AV_M8_ + MV_M2_ + AV_T6_ + AV_M2_ + MV_M8_ + MV_T6_ + MV_M1_ + AV_M1_ + AV_M6_ + MV_T1_ + MV_M6_ + AV_T1_ + AV_M3_ + MV_M3_ + AV_T8_ + MV_T8_ + MV_T3_ + AV_T3_ + MV_T7_ + AV_T5_ + MV_T4_ + AV_T4_ + AV_T7_ + MV_T2_ + MV_T5_ + AV_T2_	87.5	75

**Table 4:** Classification ability comparison.

Method	Classification Accuracy (%)
RF	82.5
PNN	65
PCA-RF	82.5
PCA-PNN	77.5
VIP-RF	92.5
VIP-PNN	90
